# Resmetirom in the Management of Metabolic Dysfunction-Associated Steatotic Liver Disease and Steatohepatitis

**DOI:** 10.3390/life15081306

**Published:** 2025-08-18

**Authors:** Fares Jamal, Amani Elshaer, Nour B. Odeh, Mayar H. Alatout, Tala Shahin, Astin R. Worden, Hashem N. Albunni, Blanca C. Lizaola-Mayo, Channa R. Jayasekera, David M. H. Chascsa, Hugo E. Vargas, Bashar A. Aqel

**Affiliations:** 1 Department of Hematology and Oncology, Mayo Clinic, Phoenix, AZ 85259, USA; 2 Department of Gastroenterology and Hepatology, Mayo Clinic, Phoenix, AZ 85259, USA; 3 Department of Cardiovascular Medicine and Surgery, Mayo Clinic, Phoenix, AZ 85054, USA; 4 Department of Colorectal Surgery, Mayo Clinic, Phoenix, AZ 85054, USA; 5 Department of Medicine, Mayo Clinic, Phoenix, AZ 85054, USA; 6 Department of Medicine, Indiana University, Indianapolis, IN 46202, USA

**Keywords:** resmetirom, MASLD, MASH, liver fibrosis, steatosis, obesity

## Abstract

Metabolic dysfunction-associated steatotic liver disease (MASLD) and its progressive form, metabolic dysfunction-associated steatohepatitis (MASH), are among the most prevalent causes of chronic liver disease worldwide, closely linked to the global rise in overweight and obesity, type 2 diabetes, and metabolic syndrome. Until recently, treatment options were limited to lifestyle interventions, with no approved pharmacologic therapies. Resmetirom, a liver-directed, selective thyroid hormone receptor beta (THR-β) agonist, is a promising disease-modifying agent that targets hepatic lipid metabolism, inflammation, and fibrosis. Clinical trials, including the pivotal phase 3 MAESTRO-NASH and MAESTRO-NAFLD-1 trials, have demonstrated significant improvements in liver fat reduction, lipid profiles, histological endpoints, and health-related quality of life, with a favorable safety profile. In March 2024, the FDA granted accelerated approval of resmetirom for adults with MASH and moderate-to-advanced fibrosis, marking a significant milestone in MASLD treatment. This review discusses the mechanistic rationale, preclinical data, and pivotal clinical trial outcomes supporting resmetirom’s role in MASLD/MASH management, while outlining ongoing research needs, including long-term safety, pediatric evaluation, and biomarker development for optimized patient selection.

## 1. Introduction

Metabolic dysfunction-associated steatotic liver disease (MASLD) has become a leading cause of chronic liver disease worldwide, currently affecting nearly one-third of the world’s population [1]. This condition is associated with an increased risk of cirrhosis, hepatocellular carcinoma, and adverse cardiovascular events [2,3]. MASLD affects approximately 38% of adults globally, with regional prevalence ranging from 20 to 80% depending on obesity and diabetes rates [4]. Of those with MASLD, approximately 20–30% progress to MASH, and among MASH patients, 10–15% develop advanced fibrosis or cirrhosis over time [5,6]. MASLD/MASH is now a leading indication for liver transplantation in many regions, and is associated with increased cardiovascular morbidity and all-cause mortality [5]. These trends parallel the rising prevalence of obesity and type 2 diabetes, underscoring the growing public health burden [5]. As case numbers rise, many healthcare systems are struggling to stay on track with early identification and long-term management. Screening programs remain limited, especially in underserved areas, and efforts to address modifiable risk factors through public health initiatives have been inconsistent or underfunded.

First-line treatment for MASLD primarily involves lifestyle interventions such as weight reduction, increased physical activity, and dietary changes [7]. While these strategies can be effective, sustaining long-term lifestyle modifications is difficult to achieve for most patients. Pharmacologic treatment options have historically been limited, and as the global prevalence of both MASLD and its progressive form, MASH, continues to rise, the need for effective targeted therapies has become increasingly urgent. Resmetirom, an oral, once-daily thyroid hormone receptor beta (THR-β) selective agonist, has been developed as a promising pharmacologic agent to address this therapeutic gap [8]. In contrast to strategies that focus solely on lifestyle changes, resmetirom directly addresses the metabolic and fibrotic pathways of the liver. By restoring THR-β activity, which is impaired in MASLD, resmetirom reduces liver fat accumulation and inflammation, potentially altering disease progression, with minimal systemic effects because of its liver-targeted action [9,10,11]. This review summarizes the pharmacologic rationale for resmetirom, along with its mechanism of action, safety profile, supporting clinical data, and future directions in MASLD/MASH management.

## 2. Pathobiology and Mechanism of Action

Progression from steatosis to steatohepatitis and fibrosis reflects coordinated crosstalk among hepatocytes, liver immune cells, liver sinusoidal endothelial cells (LSECs), and hepatic stellate cells (HSCs), with additional inputs from the gut and adipose tissue [12]. Lipotoxic, mitochondrially stressed hepatocytes release damage-associated molecular patterns (DAMPs) and lipid species that activate Kupffer cells and recruit monocyte-derived macrophages via chemokines (e.g., CCL2), amplifying pro-inflammatory cytokines (TNF-α, IL-1β, IL-6) and cytotoxic lymphocyte responses (CD8+, Th1/Th17) that further injure hepatocytes [12]. In parallel, LSECs undergo capillarization (loss of fenestrations, reduced nitric oxide), secrete CXCL12, PDGFs, and TGF-β, and promote HSC activation [12,13]. Quiescent HSCs, stimulated by mediators from injured hepatocytes (oxidized lipids, TGF-β), macrophages (TGF-β, PDGFs), and dysfunctional LSECs, transdifferentiate into myofibroblasts that deposit type I/III collagen and remodel the extracellular matrix, locking in a self-reinforcing injury–inflammation–fibrosis loop [13]. Extrahepatic axes intensify this biology: dysbiosis and increased gut permeability deliver LPS and bile acid derivatives to the liver (altering FXR/TGR5 signaling), while adipose insulin resistance increases flux of free fatty acids and shifts adipokines (leptin↑, adiponectin↓), worsening steatosis and sensitizing hepatocytes and HSCs to fibrogenic cues [14]. This integrated circuitry provides the rationale for targeting hepatocellular metabolic pathways via selective THR-β agonism.

Thyroid hormone receptors (THRs) are nuclear receptors consisting of two main isoforms: alpha (THR-α) and beta (THR-β) [15]. While THR-α is predominantly expressed in the heart and bone, THR-β is highly expressed in the liver [15]. In a healthy liver, triiodothyronine (T3) binds to THR-β to stimulate mitochondrial fatty acid β-oxidation through autophagy, especially lipophagy [16]. Thyroid hormone also suppresses hepatic lipogenesis, regulates cholesterol synthesis and clearance, and modulates bile acid metabolism. These coordinated actions maintain lipid homeostasis and prevent steatosis [17]. In MASLD and MASH, THR-β signaling is impaired, leading to hepatic lipid accumulation, mitochondrial dysfunction, and disease progression [18]. This dysfunction is further worsened by altered thyroid hormone metabolism leading to reduced T3 and increased reverse T3 (rT3) levels [15,19]. This impairment promotes lipotoxicity and oxidative stress, amplifying hepatocyte injury and inflammatory signaling (e.g., TGF-β), which drives hepatic stellate cell activation and fibrosis progression [18].

**Figure 1:**
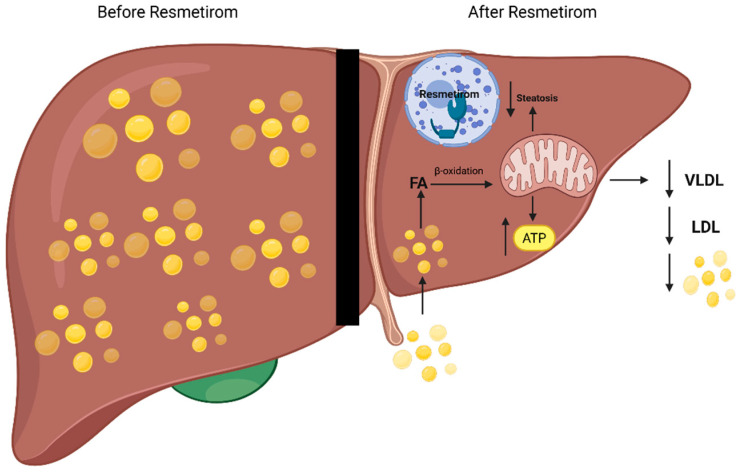
Hepatic effects of resmetirom in patients with MASLD/MASH. This schematic illustrates the efficacy of resmetirom in reducing hepatic steatosis in patients with MASLD/MASH. On the left, the liver, before treatment, shows an intracellular accumulation of lipids (yellow droplets). On the right, resmetirom enters the hepatocyte and binds to thyroid hormone receptor beta (THR-β) in the nucleus. This leads to enhanced uptake of lipids into the liver, which are broken down to fatty acids (FA), leading to increased mitochondrial β-oxidation. As a result, hepatic steatosis is reduced, adenosine triphosphate (ATP) production is increased, and very-low-density lipoprotein (VLDL), low-density lipoprotein (LDL), and serum lipid levels are decreased.

To address these defects, resmetirom was developed as a highly selective THR-β agonist. It is an orally administered compound with a 28-fold higher selectivity for THR-β over THR-α, enabling liver-specific activation without off-target effects on the heart or bone [10]. By selectively targeting THR-β in hepatocytes, resmetirom enhances mitochondrial fatty acid β-oxidation and reduces hepatic lipid accumulation [20]. It is also hypothesized to reduce hepatic very-low-density lipoprotein (VLDL) secretion, thereby lowering circulating triglycerides and low-density lipoprotein (LDL) cholesterol [21]. Overall, resmetirom acts to decrease hepatic steatosis, improve lipid metabolism, and increase energy expenditure without systemic thyroid hormone-related side effects [22]. Other liver-directed THR-β agonists, including VK2809 and ASC41, are currently in phase II–III clinical trials for MASLD and MASH [23]. Figure 1 summarizes the proposed mechanism by which resmetirom improves hepatic lipid metabolism in MASLD/MASH.

## 3. Preclinical Trials

Animal and in vitro studies have consistently demonstrated resmetirom’s efficacy in activating hepatic THR-β, enhancing lipid metabolism, suppressing inflammatory pathways, and promoting antifibrotic remodeling, processes that are central to reversing MASH pathology [24]. Multiple rodent studies show that resmetirom markedly reduces hepatic triglyceride accumulation, improves liver histology, and nearly normalizes serum total cholesterol while lowering triglycerides [25,26]. In diet-induced-obesity (DIO) mouse models of MASH, resmetirom improves the NAFLD activity score, significantly lowering serum total cholesterol and triglycerides [25]. Resmetirom has also demonstrated the ability to modulate fibrogenic pathways by downregulating key fibrogenic markers, including Col1a1 (collagen type I alpha 1 chain, a significant component of fibrotic extracellular matrix) and Acta2 (α-SMA, alpha-smooth muscle actin, a marker of activated hepatic stellate cells, which drive fibrogenesis), at both the mRNA and protein levels [25]. Furthermore, resmetirom has been found to suppress pro-inflammatory and pro-fibrotic mediators, including transforming growth factor-beta (TGF-β). It reduces lipogenesis by downregulating lipogenic genes encoding fatty acid synthase (FASN) and acetyl-CoA carboxylase 1 (ACC1), both of which are often elevated in MASLD [27,28].

Advanced in vitro models further support these findings. In a humanized liver-on-chip (LoC) MASLD model, resmetirom prevented intracellular lipid accumulation in primary hepatocytes and reduced pro-inflammatory cytokines such as IL-10, CCL2/MCP-1, and IFN-γ. It also downregulated Col1a1 and α-SMA, mirroring its antifibrotic effects observed in vivo [29]. In summary, resmetirom demonstrates robust preclinical efficacy in targeting the key pathophysiologic mechanisms of MASH, including steatosis, inflammation, and fibrogenesis.

## 4. Clinical Trials

Several clinical studies have evaluated the safety and efficacy of resmetirom (summarized in Table 1). The first was a multicenter, randomized, double-blinded phase 2 trial conducted at 25 institutions in the United States to evaluate resmetirom’s effect on MASH [21]. In a 2:1 ratio, a total of 125 patients were randomized to receive either 80 mg of resmetirom (*n* = 84) or a placebo (*n* = 41) once daily for 36 weeks. The primary endpoint that was investigated was the change in hepatic fat fraction at week 12, assessed by magnetic resonance imaging proton density fat fraction (MRI-PDFF). As for the secondary endpoints, the study included changes recorded at week 36, histological improvements, and safety outcomes.

Resmetirom was shown to significantly reduce hepatic fat compared to the placebo at both week 12 (−32.9% vs. −10.4%; least squares mean difference −22.5%, 95% CI −32.9 to −12.2; *p* < 0.0001) and week 36 (−37.3% vs. −8.5%; difference −28.8%, 95% CI −42.0 to −15.7; *p* < 0.0001). Histological analyses revealed a trend toward greater resolution of MASH and improvements in fibrosis with resmetirom, though the study was not powered by these endpoints.

Adverse effects were mostly mild or moderate and balanced between groups, with a higher incidence of transient mild diarrhea and nausea in the resmetirom group. The conclusion drawn was that resmetirom significantly reduces hepatic fat in MASH patients and is generally well tolerated, supporting further investigation in larger phase 3 trials to establish its safety and an association between histological and non-invasive outcomes, leading to the MAESTO-NASH phase 3 trial. 

The MAESTRO-NASH trial is a crucial phase 3, double-blind, randomized, placebo-controlled study that is currently ongoing [30]. It serves to evaluate the efficacy and safety of resmetirom in adults with biopsy-confirmed MASH and liver fibrosis stages F1B, F2, or F3. This trial is being conducted in 15 countries, involving 245 sites. A total of 966 patients are enrolled (322 in the 80 mg resmetirom group, 323 in the 100 mg group, and 321 in the placebo group). Patients were randomized in a 1:1:1 ratio to receive, once a day, 80 mg of resmetirom, 100 mg of resmetirom, or a placebo. The inclusion criteria required patients to be ≥18 years old with three metabolic risk factors, histological evidence of MASH or MASLD, a stable weight, and an activity score ≥4. The majority of the participants (65%) had advanced fibrosis (stage F3).

At 52 weeks, the primary endpoints were MASH resolution (defined by the researchers as a hepatocellular ballooning score of 0, a lobular inflammation score of 0 or 1, and a reduction in the MASLD activity score by ≥2 points, without fibrosis worsening) and an improvement in liver fibrosis by at least one stage (with no worsening of MASLD activity score). At 52 weeks, MASH resolution without any fibrosis worsening was achieved in 25.9% in the 80 mg resmetirom group and 29.9% in the 100 mg resmetirom group, versus 9.7% in the placebo group (*p* < 0.001). Improvement in fibrosis by at least one stage with no worsening of MASLD activity score occurred in 24.2% (80 mg) and 25.9% (100 mg) of resmetirom-treated patients, versus 14.2% of those receiving the placebo (*p* < 0.001 for both comparisons). Additionally, at week 24, resmetirom resulted in significant reductions in LDL cholesterol of 13.6% (80 mg) and 16.3% (100 mg), compared to 0.1% for those receiving the placebo (*p* < 0.001).

The incidence of serious adverse events was 10.9% (80 mg), 12.7% (100 mg), and 11.5% (placebo), corresponding to absolute differences of −0.6% (80 mg vs. placebo) and +1.2% (100 mg vs. placebo). Diarrhea and nausea were more frequent in the resmetirom arms. Overall, the study concluded that resmetirom doses of both 80 mg and 100 mg demonstrated superiority over the placebo in achieving histological endpoints of MASH resolution and fibrosis improvement at 52 weeks, with a generally favorable safety profile, supporting its potential utilization as a disease-modifying therapy for MASH with fibrosis.

Another multicenter, randomized trial, the MAESTRO-NAFLD-1 trial [8], was conducted to evaluate the metabolic efficacy and safety of resmetirom in adults with MASLD and presumed MASH, using a non-invasive diagnostic criterion. The study was conducted between 16 December 2019 and 13 December 2020. It involved 1143 participants: 325 were assigned to resmetirom 100 mg, 327 to resmetirom 80 mg, 320 to a placebo, and 171 to open-label resmetirom 100 mg. The primary endpoint was the incidence of treatment-emergent adverse events (TEAEs) at 52 weeks. Key secondary endpoints included changes in LDL cholesterol, triglycerides, apolipoprotein B (apoB), and the hepatic fat fraction, measured by MRI-PDFF at 16 and 52 weeks, as well as liver stiffness, assessed by transient elastography at 52 weeks.

The results indicated that 86.1% of participants in the 100 mg resmetirom group, 88.4% in the 80 mg group, and 81.8% in the placebo group experienced at least one TEAE over the 52 weeks. The most common TEAEs were mild gastrointestinal symptoms, particularly diarrhea (100 mg: 23.1%, 80 mg: 24.8%, placebo: 12.5%) and nausea (100 mg: 13.8%, 80 mg: 13.4%, placebo: 8.4%). Serious adverse events were comparable across the groups (100 mg: 7.4%, 80 mg: 6.7%, placebo: 7.2%), and the rate of discontinuations due to adverse events was low (100 mg: 4.6%, 80 mg: 4.6%, placebo: 2.7%). Notably, there were no instances of drug-induced liver injury or deaths attributed to the study drug.

For metabolic efficacy, at week 24, the least-squares mean differences from the placebo in LDL-C were as follows: −11.1% for the 80 mg dose and −12.6% for the 100 mg dose. For apoB, the differences were −15.6% (80 mg) and −18.0% (100 mg). In terms of triglycerides, the reductions were −15.4% for the 80 mg group and −20.4% for the 100 mg group; all of these differences were statistically significant (*p* < 0.0001). The hepatic fat fraction, measured by MRI-PDFF, decreased at week 16 by −34.9% in the 80 mg group and −38.6% in the 100 mg group compared to the placebo group. By week 52, these reductions were −28.8% (80 mg) and −33.9% (100 mg), with *p* < 0.0001.

Liver stiffness, assessed via transient elastography, decreased by −1.02 kPa (80 mg) and −1.70 kPa (100 mg) compared to in the placebo group. In total, 77.4% of patients in the double-blind arms completed the study, with discontinuation rates not differing significantly between groups. The trial population was high-risk, characterized by significant metabolic comorbidities. However, the study was limited because most patients were in the early stages of fibrosis and utilized non-invasive, rather than histologic, endpoints.

Overall, the MAESTRO-NAFLD-1 trial demonstrated that resmetirom is safe and well tolerated in patients with MASLD and presumed MASH. The study showed significant improvements in atherogenic lipids and hepatic steatosis, supporting the continued development of resmetirom for metabolic liver disease.

Another phase 2, double-blinded, multi-center, randomized, placebo-controlled trial was conducted in order to evaluate the impact of resmetirom on health-related quality of life (HRQL) and hepatic fat reduction in adults with biopsy-proven MASH and a hepatic fat fraction of ≥10% as measured by MRI-PDFF [31].

A total of 125 patients were enrolled in the study, with a mean age of 50 ± 11 years. The patients were randomized into two groups: 84 received 80 mg of resmetirom daily, while 41 received a placebo for a duration of 36 weeks. The primary endpoint of the study was any change in health-related quality of life (HRQL), which was assessed using the Short Form-36 (SF-36) survey. Secondary endpoints included reductions in hepatic fat, changes in the MASLD Activity Score, and histological improvements observed through liver biopsy.

At baseline, the HRQL scores of the patients were comparable to those of the general population. By week 12, patients receiving resmetirom showed significant improvements in pain (reported as musculoskeletal, abdominal, or generalized aches), and these improvements continued through week 36, while the placebo group did not exhibit any positive changes. Patients who achieved a reduction of 30% or more in their hepatic fat fraction by week 12 also demonstrated greater enhancements in physical functioning and overall physical component scores by week 36, regardless of their baseline characteristics.

Additionally, those who experienced histological improvements in MASH and fibrosis also benefitted in terms of HRQL.

The study concluded that resmetirom significantly reduced hepatic fat and led to meaningful improvements in quality of life for patients with MASH. This was particularly evident among those who achieved a substantial decrease in liver fat and showed histological improvement, indicating the need for further evaluation in larger clinical trials.

## 5. Guidelines

In March 2024, the Food and Drug Administration (FDA) granted accelerated approval for resmetirom to become the first drug approved for adults with MASH and moderate-to-advanced hepatic fibrosis in the United States [32]. Following this, the American Association for the Study of Liver Diseases (AASLD) updated its clinical guidance to incorporate resmetirom, stressing the importance of continued real-world data collection and long-term studies to define better its role in managing MASLD and MASH [30]. However, approvals outside of the United States are pending as of August 2025.

According to AASLD guidance, adults with biopsy-confirmed MASH and moderate-to-advanced fibrosis (F2–F3), or non-invasive test results strongly suggestive of these stages when biopsy is not feasible, are eligible for resmetirom therapy [11]. The AASLD further advises against the use of resmetirom in patients with cirrhosis, whether compensated or decompensated; uncontrolled active liver diseases (such as autoimmune hepatitis or primary biliary cholangitis); or ongoing significant alcohol consumption. Further uncontrolled thryoid disease is considered an absolute contraindication. These guideline-based criteria and dose adjustments are summarized in Table 2 [11].

Before initiating therapy, baseline evaluations should include liver stiffness measurement (transient elastography or MR elastography), a comprehensive metabolic panel with liver enzymes, a lipid profile, thyroid function tests, and pregnancy testing. Cardiovascular risk assessment and a review of concomitant medications, particularly statins and CYP2C8 inhibitors, are also recommended [11].

During therapy, patients should be monitored at approximately 3, 6, and 12 months with repeat liver enzymes, lipid profiles, and thyroid function tests to assess safety and detect drug-related adverse effects. Efficacy can be assessed through non-invasive fibrosis testing (e.g., elastography) and hepatic fat quantification (MRI-PDFF or CAP score) at 12 months, alongside clinical evaluation for metabolic risk factor control [11].

A ≥30% reduction in hepatic fat content on MRI-PDFF is associated with improved histologic outcomes and is considered a favorable response, but this observation alone is not sufficient to define treatment success. The AASLD specifically notes that while a ≥30% reduction in MRI-PDFF after 1 year of resmetirom therapy is associated with improved histologic outcomes, the positive predictive value for histologic response is less than 50%, and thus this is insufficient for it to be used as a sole criterion for efficacy. Changes in CAP have not been validated as predictors of histologic response and are not recommended as a primary monitoring tool. An improvement in liver stiffness by ≥20% (MRE) or ≥25% (VCTE) is considered clinically meaningful, but these thresholds are not definitive for histologic response, and VCTE in particular may overestimate response. Normalization or significant improvement in aminotransferases (e.g., ALT reduction by ≥17 U/L or to ≤40 U/L and by ≥30% from baseline) is considered supportive, but not definitive. Favorable lipid profile changes are expected with resmetirom, but are not used as primary efficacy endpoints. The AASLD recommends a holistic assessment incorporating changes in MRI-PDFF (if available), liver stiffness (MRE/VCTE), aminotransferases, and clinical context to determine ongoing benefits from resmetirom, rather than relying on a composite of the specific criteria. If there is no meaningful biochemical or imaging improvement after 12 months, as determined by a holistic assessment of MRI-PDFF, liver stiffness, aminotransferases, and clinical context, resmetirom therapy should be discontinued due to futility [11]. In practice, response assessment often involves interpreting small, sometimes inconsistent changes across different tests. Clinical judgment remains key, particularly when test results conflict or when symptoms evolve. A patient’s overall trajectory often says more than any single measurement.

## 6. Future Implications and Research Gaps

While the development of therapeutics for MASLD and MASH, including resmetirom, has shown considerable promise, several research gaps and concerns regarding their clinical application remain. Further research is needed to evaluate the safety and efficacy of resmetirom in pediatric populations under 18 years of age. In addition, important research gaps remain, including the limited representation of diverse ethnic and geographic populations in pivotal trials and the lack of data in patients with cirrhosis or those with very-early-stage disease. Another key priority is the identification and validation of predictive biomarkers and clinical indicators, such as MRI-PDFF, VCTE, and MRE, that can reliably determine therapeutic response in MASH. The role of germline mutations in MASH pathogenesis and their potential influence on resmetirom efficacy also warrants exploration. Moreover, studies assessing the long-term effects of resmetirom on bone and cardiac health are essential to ensure its safety beyond hepatic targets. In addition, the interaction between resmetirom and glucagon-like peptide-1 (GLP-1), dual GLP-1/glucose-dependent insulinotropic polypeptide (GIP), or triple agonists warrants further investigation. Finally, it remains to be determined whether early intervention with resmetirom in patients with MASLD could prevent progression to MASH, as well as the optimal treatment duration, cost-effectiveness in real-world settings, and strategies for sequencing or combining resmetirom with other MASLD therapies.

## 7. Conclusions

Resmetirom represents a promising advancement in the treatment of MASH, activating liver-specific THR-β. Clinical trials have shown meaningful improvements in liver fat reduction, biomarkers of fibrosis, and lipid metabolism, with a favorable safety profile. As the first FDA-approved medication for MASH with fibrosis, resmetirom marks a significant step forward in the management of metabolic liver disease. However, unlike GLP-1 receptor agonists, resmetirom does not induce weight loss, a feature increasingly associated with fibrosis regression in emerging data. Long-term safety, efficacy across diverse populations, and its role in combination therapy remain areas for future research. Ongoing post-marketing surveillance and real-world studies will be critical to establish its long-term clinical utility.

## Figures and Tables

**Table 1:** Key findings from clinical trials of resmetirom usage in patients with MASLD and MASH.

Trial Name	Phase	Population	Sample Size	Design	Primary Endpoint	Key Findings
MAESTRO-NASH (NCT03900429) [30]	III	Biopsy-confirmed MASH and liver fibrosis stages: F1B, F2, or F31	966	Randomized, double-blind, placebo-controlled	MASH resolution and fibrosis improvement at week 52	Significant MASH resolution and fibrosis improvement vs. placebo
MAESTRO-NAFLD-1 (NCT04197479) [8]	III	MASLD/presumed MASH via non-invasive testing	1143	Randomized, double-blind, placebo-controlled	TEAEs at 52 weeks; metabolic and imaging endpoints	Improvements in LDL-C, apoB, TGs, and hepatic fat; good safety profile
HRQL Study (NCT02912260) [31]	II	Biopsy-proven MASH and a hepatic fat fraction of ≥10% as measured by MRI-PDFF	125	Randomized, double-blind, placebo-controlled	HRQL change (SF-36); hepatic fat reduction	Improvements in HRQL and liver fat in responders; histological benefit tied to QOL

Abbreviations: apoB: apolipoprotein B, HRQL: health related quality of life, LDL-C: low density lipoprotein cholesterol, MASH: metabolic dysfunction-associated steatohepatitis, MASLD: metabolic dysfunction-associated steatotic liver disease, MRI-PDFF: magnetic resonance imaging proton density fat fraction, QOL: quality of life, TEAE: treatment-emergent adverse event, TG: triglycerides.

**Table 2:** AASLD-backed candidate criteria for resmetirom use in MASLD/MASH.

Category	Criteria
Indication	Adults with biopsy-confirmed MASH and moderate-to-advanced fibrosis (F2–F3)
Resmetirom Dosing	Dosing: Dose of 80 mg/day for weight <100 kg. Dose of 100 mg/day for weight ≥100 kg. Dose of 60–80 mg/day if on moderate CYP2C8 inhibitor (e.g., clopidogrel). Avoid use with strong CYP2C8 inhibitors (e.g., gemfibrozil).
Statin Dosing	Max. statin dose if co-administered: ○ Atorvastatin: 40 mg/day. ○ Pravastatin: 40 mg/day. ○ Rosuvastatin: 20 mg/day. ○ Simvastatin: 20 mg/day.
Contraindications	Cirrhosis (compensated or decompensated). Autoimmune liver diseases (e.g., AIH, PBC). Significant alcohol use (>20g/day in women or >30g/day in men). Uncontrolled thyroid disease.

AIH: autoimmune hepatitis, CYP2C8: cytochrome P450 2C8 enzyme, F2–F3: fibrosis stage 2 to 3, MASH: metabolic dysfunction-associated steatohepatitis, PBC: primary biliary cholangitis.
